# In sito galanthamine extraction during the cultivation of *Leucojum aestivum* L. shoot culture in two‐phase bubble column cultivation system

**DOI:** 10.1002/elsc.201900106

**Published:** 2019-09-19

**Authors:** Ivan Ivanov, Strahi Berkov, Atanas Pavlov, Vasil Georgiev

**Affiliations:** ^1^ University of Food Technologies Plovdiv Bulgaria; ^2^ Institute of Biodiversity and Ecosystem Research Sofia Bulgaria; ^3^ Laboratory of Applied Biotechnology The Stephan Angeloff Institute of Microbiology Bulgarian Academy of Sciences Plovdiv Bulgaria

**Keywords:** absorption resin, bubble‐column cultivation system, galanthamine, shoot cultures, summer snowflake

## Abstract

Two‐phase bioreactor cultivation system was developed and applied for in sito recovery of extracellular galanthamine during the cultivation of *Leucojum aestivum* L. shoot culture in a modified column bioreactor system. The inclusion of an external circulation column with adsorbent resin Amberlite XAD‐4 as a second phase, on the 21st day of the beginning of cultivation resulted in 1.25 folds increase in biomass accumulation and maximal amounts of accumulated galanthamine of 6 mg/L (3.1 mg/L intracellular and 2.9 mg/L extracellular). It was demonstrated that the inclusion of a second phase at the cultivation of the *L. aestivum* shoot culture in a bubble column bioreactor with internal sections redirected the alkaloid metabolism to galanthamine synthesis and inhibits the synthesis of hemanthamine and lycorine type alkaloids. Our research demonstrated that the application of the two‐phase cultivation systems could be an important tool to increase the yields of valuable secondary metabolites in plant tissue culture‐based bioprocess.

**Abbreviation**
MS: Murashige and Skoog nutrient medium
TIC: total ion current

## 1 INTRODUCTION

Amaryllidaceae alkaloid galanthamine is a long‐acting, reversible, selective, and competitive acetylcholinesterase inhibitor that crosses the blood–brain barrier 1. In the market, it is available as well a hydrobromide salt under the names of Razadine^®^ (formerly Reminyl^®^) and Nivalin^®^. Galanthamine has been widely used for treatment of early‐ to mid‐stage Alzheimer's disease, poliomyelitis, and other neurological diseases 2.

Nowadays, plants remain the main source of galanthamine; nevertheless, its chemical synthesis has been achieved 3. The main sources for the commercial production of the galanthamine are the plant species called daffodils (*Narcissus*) and summer snowflake (*Leucojum aestivum* L.) 4, 5. In the three decades, many natural *L. aestivum* habitats have shrunk or fallen under threat due to the growing demand by the pharmaceutical companies and, the biosynthesis of galanthamine by plant in vitro systems has been considered as a prospective alternative approach for sustainable production of this valuable alkaloid. Even galanthamine, produced by plant cell cultures, is more expensive than chemically synthesized one, it is considered as a natural product and is free of unwanted contaminating residues, which makes it safer for medicinal application. It is well known that many species from Amaryllidaceae family synthesized galanthamine. Nevertheless, the research on its in vitro production is focused only on *Narcissus cofusus* Pugsley and *L. aestivum*. The first scientific manuscripts on the biosynthesis of galanthamine by *L. aestivum* in vitro systems appeared during the first decade of this century 6, 7, 8, 9. These investigations lead to conclusion that the cell differentiation is essential for the galanthamine biosynthesis 9, 10. It was established that shoot cultures had higher potential to synthesize this alkaloid as compared to hairy roots described by Diop and coworkers 11. Yields of galanthamine, produced by shoot culture of *L. aestivum*, were strongly influenced from illumination 9, nutrient medium composition 12, and addition of elicitors 13, 14, 15, 16, 17. Additionally, our group proposed a modified formulation of the nutrient medium composition for maximal biosynthesis of galanthamine by *L. aestivum* shoots cultivated under submerged conditions 9.

At the moment, there are few papers describing bioreactor cultivation of *L. aestivum* soot cultures for the galanthamine production. Galanthamine production processes based on temporary immersion technique have been investigated 14, 17, 18. However, temporary immersion cultivation systems are not appropriate for the development of commercial production process due to well know difficulties with inoculation of bioreactors and high labor costs 19. Based on understanding, bioreactor design is essential for the success of the industrial implementation of plant biotechnologies; a process of galanthamine, production by *L. aestivum* shoot culture in an illuminated bubble‐column bioreactor with internal sections, was developed 20 in the laboratory. It was established that under optimal conditions for galanthamine biosynthesis over 40% of the biosynthesized alkaloid were extracellular. Based on this result, we noted that the two‐phase process for in situ extraction of secreted galanthamine could be a prospective technological decision to increase the effectiveness of the modified column bioreactor. Based on our previous experience, we decided to use an adsorption resins with low ion exchange capacity to realize such two‐phase cultivation system.

In the present study, for the first time, we described the process of in sito galanthamine extraction during the cultivation of *Leucojum aestivum* shoot culture in a bioreactor system.

## 2 RESULTS AND DISCUSSION

The *Leucojum aestivum* shoot cultures were initiated by planting previously obtained calli from filaments of anthers on Murashige and Skoog nutrient medium (MS) supplemented with 30 g/L sucrose, 5.5 g/L ‘‘Plant agar’’ (Duchefa, The Netherlands), 1.15 mg/L 1‐naphthylacetic acid (Duchefa, The Netherlands), and 2 mg/L 6‐benzylaminopurine (Duchefa, The Netherlands) using the protocol described previously 9. Single shoots, differentiated from callus tissue, were propagated to give a raise of homogenous lines. The cultivation of the established shoot clumps was carried out at 26°C under illumination 16:8 h (light:dark) at a light intensity of 110 µmol/(m^2^ s) ‐ SYLVANIA Gro‐Lux fluorescent lamp (F18W/GRO‐LUX). The best growing and glanthamine producing shoot line was selected for further experiments. It is subcultivated every 28 days under the same conditions for more than five years.

**PRACTICAL APPLICATION**

In our previous investigations we found that *Leucojum aestivum* L. shoot culture biosynthesized extracellular galanthamine and related compounds, whereas lycorine and haemanthamine‐type compounds were accumulated intracellularly. In this manuscript, we describe in sito extraction of galanthamine during the cultivation of *L. aestivum* shoot culture in a new developed two‐phase bubble column cultivation system based on previously developed glass‐column bioreactor with internal sections. The presented results allowed more effective cultivation of shoot cultures that could be the base for the development of large‐scale galanthamine production processes.

**Figure 1:**
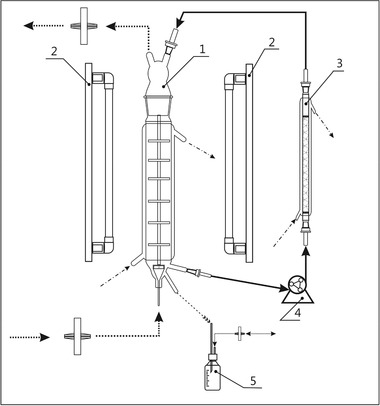
Schematic diagram of two‐phase bubble column cultivation system: 1. bubble column with internal sections; 2. fluorescent lamps; 3. column filled with adsorption resin; 4. circulation pump; 5. sampling

For bioreactor cultivation of *L. aestivum* shoots, a bubble‐column bioreactor with internal section previously described from Georgiev and co‐workers was used 20. A glass column containing second phase (10 g of adsorption resin Amberlite XAD‐4^®^, preliminary activated following protocol of the producer) was attached to the bubble‐column bioreactor (Figure 1). The cultivation was performed in the bioreactor filled with 1 L optimized MS 12, under illumination 16:8 h (light:dark) with light intensity of 110 µmol/(m^2^ s)–SYLVANIA Gro‐Lux fluorescent lamp (F18W/GRO‐LUX), at 22°C temperature of cultivation and constant flow rate of inlet air of 18 L/(L h) 12. The second phase circulation contour was connected to the cultivation system on 14th, 21st, and 28th days from the beginning of cultivation by using peristaltic pump (flow rate 1 L/h). In all experiments, the bioreactor was inoculated with 60 g (fresh weight) of shoot culture grown for 28 days on solid medium as described above.

Extraction of intracellular, extracellular, and adsorbed on the resin alkaloids followed previously described procedure 20, 21.

Analyses of the accumulated dry biomass and the GI were performed following the methods described by Georgiev et al. 20.

The galanthamine quantification was performed on Waters HPLC system, equipped with Waters 1525 binary pump (Milford, USA), a Dual λ Waters 2487 absorbance detector (Milford, USA) and Breeze 3.30 SPA software by the method described previously 18.

The GC‐MS analyses were performed with a Hewlett Packard 6890+/MSD 5975 (Hewlett Packard, Palo Alto, CA, USA) on HP‐5 MS column (30 m × 0.25 mm × 0.25 µm) as described previously 22. The spectra of co‐eluting chromatographic peaks were examined and deconvoluted by using AMDIS 2.6 (NIST, Gaithersburg, MD, USA) software before area integration. The contribution of each compound in the extracts is shown as a percentage of the total ion current (TIC). The alkaloids were identified by comparing their mass spectral fragmentations with those of standard references from NIST 08 database (NIST Mass Spectral Database, PC‐Version 5.0 (2008), National Institute of Standardization and Technology, Gaithersburg, MD, USA) and/or as described literature data 23, 24.

The area of the GC‐MS peaks depends not only on the concentration of the corresponding compounds but also on the intensity of their mass spectral fragmentation. The presented data does not express absolute values (do not represent a true quantification) but can be used for comparison of the samples, obtained during different cultivation regimes.

The results presented in the study have been summarized from two independent experiments, repeated twice. The software package MINITAB 14 was used for the assessment of the obtained experimental results.

The two‐phase bubble column cultivation system (Figure 1) was developed on the basis of previously described illuminated bubble‐column bioreactor with internal sections 20 through attaching external circulation contour with a column filled with adsorption resin Amberlite XAD‐4.

A basic parameter that should be optimized during two‐phase bioreactor cultivation of plant in vitro systems is the time of connection of the external circulation contour. On this basis, the cultivation of *L. aestivum* shoot culture in the developed two‐phase cultivation system was investigated with the inclusion of the second phase on the 14th, 21st, and 28th days of the beginning of the cultivation process. Cultivation was carried out at a temperature of 22°C and a flow rate of inlet air of 18 L/(L h) that were previously optimized 20.

**Figure 2:**
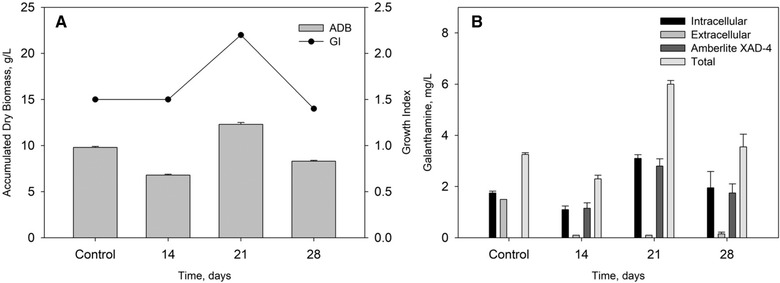
Accumulated dry biomass, growth index (A), and biosynthesized galanthamine (B) by *L. aestivum* shoot culture during its cultivation in two‐phase bubble column cultivation system

Inclusion of the second phase in the cultivation process, on the 21st day of the beginning of the cultivation, showed that the accumulated biomass increased 1.25 times as compared to the control cultivation (without adsorption resin) (9.8 g/L) and reached 12.3 g/L. The Growth Index, also increased (from 1.5 in the control cultivation to 2.2 in the cultivation with the second phase included, 1.46 times) (Figure 2). The other two regimes of adding a second phase do not affect the biomass accumulation. The results achieved demonstrated that the inclusion of the second phase to the cultivation process of *L. aestivum* shoots in bubble column bioreactor with internal sections, stimulate the growth of the plant tissues. This could be because of the adsorption and elimination of extracellular metabolites (mainly alkaloids and phenolic substances) that in other way could have a negative effect on shoots growth when accumulated in culture medium 25.

*L. aestivum* shoot culture accumulated maximal amounts of galanthamine during its cultivation when the second phase were included in the bioreactor system at 21st day of cultivation –6.0 mg/L (3.1 mg/L intracellular and 2.9 mg/L extracellular). This is 1.8 fold higher compare to the control cultivation, without second phase (3.3–1.8 mg/L intracellular galanthamine and 1.5 mg/L extracellular) (Figure 2). Using the second phase leaded to induction of both galanthamine biosynthesis and its secretion from the plant tissues into the culture liquid. The amount of the accumulated intracellular galanthamine at the two‐phase cultivation was 1.7 fold higher (3.1 mg/L) compared to the control (1.8 mg/L), while extracellular galanthamine increased its level 1.9 fold (2.9 mg/L) compared to the control (1.5 mg/L). It should be underlined that 96.5% of extracellular galanthamine (2.8 mg/L) was adsorbed on the Amberlite XAD‐4 (second phase) and only 0.1 mg/L was detected in the culture liquid. Achieved results clearly showed that inclusion of the second phase in the cultivation system on the 21st day of the beginning of the cultivation influenced on the equilibrium of the galanthamine between plant tissue and the extracellular environment and the plant cell synthesized galanthamine de novo and on the other hand secretion onto the culture liquid was improved significantly. When the second phase was included to the cultivation system at 28th day of the cultivation, total amount of the biosynthesized galanthamine was comparable with control cultivation −3.2 mg/L (1.1 mg/L intracellular and 2.1 mg/L extracellular galanthamine). At this case a change in the equilibrium between extracellular and intracellular galanthamine was observed. At the control cultivation the amount of extracellular and intracellular galanthamine were comparable, while with inclusion of the second phase the amount of the intracellular galanthamine is 2 fold higher than the extracellularly accumulated (Figure 2).

The maximal productivity of the cultivation system [168.6 µg/(L d)] was achieved when the second phase was included to the system on the 21st day of cultivation. This fold was higher as compared with the control cultivation. The obtained productivity [168.6 µg/(L d)] was significantly higher than the previously reported galanthamine production by *L. aestivum* line 80 shoot culture when cultivated in modified bubble column bioreactor [48.6 µg/(L d)] and in temporary immersion RITA systems [37.9 µg/(L d)] 18, 20.

The alkaloid profiles of the different components of the two‐phase cultivation systems (with inclusion of the second phase on the 21st day of cultivation) were also investigated. Results are presented in Table 1. Eight alkaloids were detected at the two‐phase cultivation and 12 at the control. The amount of the intracellular galanthamine was increased at the two‐phase cultivation. At the investigated regime of the two‐phase cultivation (inclusion of the second phase on the 21st day of cultivation), only the galanthamine was identified in the intracellular alkaloid extract. It was also the single alkaloid identified in the culture liquid of the two‐phase cultivation system. So, the inclusion of the second phase in the cultivation system directed the metabolism of *L. aestivum* shoot culture to the galanthamine. Direct precursor of galanthamine, the *N*‐demethylgalanthamine, was identified only adsorbed on the second phase. 8‐*O*‐Demethylmaritidine was identified in the intracellular alkaloid extracts with second higher concentration at the control cultivation, was not identified in the *L. aestivum* biomass cultivated in the two‐phase system. This alkaloid was identified adsorbed on the second phase (2.5% of the TIC). Vittatine also was identified only adsorbed on the second phase (2.9% of the TIC), while at the control cultivation it was determined both in the biomass and in the culture liquid (Table 1).

**Table 1:** Alkaloid profile of *L. aestivum* shoot line cultivated in modified bubble column bioreactor under standard conditions (control), and under two‐phase cultivation conditions including the circulation contour with Amberlite XAD‐4, connected on 21st day

	Control		Two‐phase cultivation		
Alkaloids	Biomass	Culture liquid	Biomass	Culture liquid	Resin
Apogalanthamine	tr.	0.1	–	–	–
Trisphaeridine	0.1	tr.	–	–	–
Galanthamine	78.8	92	100	100	90.7
*N*‐Demethylgalanthamine	3.2	Tr	–	–	0.4
Vittatine	0.2	1.5	–	–	2.9
Narwedine	0.4	4.7	–	–	2.4
8‐*O*‐Demethylmaritidine	12.9	–	–	–	2.5
Pancracine C	0.1	–	–	–	0.1
11,12‐Didehydroanhydrolycorine	–	0.1	–	–	0.1
Hamayne	3.2	1	–	–	0.9
Lycorine	0.6	0.1	–	–	–
*N*–Formylnorgalanthamine	0.3	0.4	–	–	–

tr. < 0.1% of TIC

The identified alkaloids are grouped according to their structural type in three groups ‐ galanthamine, hemanthamine and lycorine types. Galanthamine type alkaloids with para‐ortho phenol‐oxidative structure are the only type of alkaloids found in biomass and in culture liquid in the two‐phase cultivation system (Table 1). In the alkaloid extracts adsorbed to the second phase (Amberlite XAD‐4), galanthamine‐type alkaloids are more than 93% of the TIC. The alkaloids with para‐para phenol‐oxidative structure were fully adsorbed on the second phase, 6.4% of the TIC. The lycorine type alkaloids were presented in traces adsorbed on the second phase −0.1% of the TIC.

In conclusion, the addition of a second phase at the cultivation of the *L. aestivum* shoot culture in bubble column bioreactor with internal sections directed the alkaloid metabolism to galanthamine synthesis and inhibits the synthesis of hemanthamine and lycorine type alkaloids. This fact, in parallel to the achieved higher volumetric yields and productivity (the best ones ever reported in the scientific literature) of the investigated two‐phase cultivation system, are an important step in the development of commercial process. In our opinion this cultivation approach could be applied to increase the target alkaloid yields in *in vitro* cultivated galantthamine producing plant cell tissue or organ cultures and thus, could contribute to economical effectiveness and commercialization of this perspective biotechnological process.

## CONFLICT OF INTEREST

The autors have declared no conflict of interest.

## References

[1] Thomsen, T. , Bickel, U. , Fischer, J. , Kewitz, H. , Stereoselectivity of cholinesterase inhibition by galanthamine and tolerance in humans. Eur. J. Clin. Pharmacol. 1998, 39, 603–605. 10.1007/BF00316106 2095347

[2] Heinrich, M. , Teoh, H. L. , Galanthamine from snowdrop‐the development of a modern drug against Alzheimer's disease from local Caucasian knowledge. J. Ethnopharmacol. 2004, 92, 147–162. 15137996 10.1016/j.jep.2004.02.012

[3] Satcharoen, V. , McLean, N. J. , Kemp, S. C. , Camp, N. P. et al., Stereocontrolled synthesis of (‐)‐galanthamine. Org. Lett. 2007, 9, 1867–1869. 17429978 10.1021/ol070255i

[4] Parolo, G. , Abeli, T. , Rossi, G. , Dowgiallo, G. et al., Biological flora of central Europe: *Leucojum aestivum* L. Perspect. Plant Ecol. Evol. Syst. 2011, 13, 319–330.

[5] Selles, M. , Bergonon, S. , Viladomat, F. , Bastida, J. et al., Effect of sucrose on growth and galanthamine production in shoot clump cultures of *Narcissus confusus* in liquid‐shake medium. Plant Cell Tissue Organ Cult. 1997, 49, 129–136.

[6] Berkov, S. , Pavlov, A. , Iliava, M. , Burrus, M. et al., CGC/MS of alkaloids in *Leucojum aestivum* plants and there in vitro cultures. Phytochem. Anal. 2005, 16, 98–103. 15881117 10.1002/pca.824

[7] Diop, M. F. , Ptak, A. , Chretien, F. , Henry, M. et al., Galanthamine content of bulbs and in vitro cultures of *Leucojum aestivum* L. Nat. Prod. Commun. 2006, 1, 475–479.

[8] Georgieva, L. , Berkov, S. , Kondakova, V. , Bastida, J. et al., Alkaloid variability in *Leucojum aestivum* from wild populations. Z. Naturforsch. C 2007, 62, 627–635. 18069233 10.1515/znc-2007-9-1002

[9] Pavlov, A. , Berkov, S. , Courot, E. , Gocheva, T. et al., Galanthamine production by *Leucojum aestivum in vitro* systems. Process Biochem. 2007, 42, 734–739.

[10] Ptak, A. , Tahchy, A. , Wy˙zgolik, G. , Henry, M. , Laurain‐Mattar, D. , Effects of ethylene on somatic embryogenesis and galanthamine content in *Leucojum aestivum* L. cultures. Plant Cell Tissue Organ Cult. 2010, 102, 61–67.

[11] Diop, M. F. , Hehn, A. , Ptak, A. , Chretien, F. et al., Hairy root and tissue cultures of *Leucojum aestivum* L. – relationships to galanthamine content. Phytochem. Rev. 2007, 6, 137–141.

[12] Georgiev, V. , Berkov, S. , Georgiev, M. , Burrus, M. et al., Optimized nutrient medium for galanthamine production in *Leucojum aestivum* L. *in vitro* shoot system. Z. Naturforsch. C 2009, 64, 219–224. 19526716 10.1515/znc-2009-3-412

[13] El Tahchy, A. , Bordage, S. , Ptak, A. , Dupire, F. et al., Effects of sucrose and plant growth regulators on acetylcholinesterase inhibitory activity of alkaloids accumulated in shoot cultures of Amaryllidaceae. Plant Cell Tissue Organ Cult. 2011, 106, 381–390.

[14] Ptak, A. , Moranska, E. , Saliba, S. , Zielinski, A. et al., Elicitation of galanthamine and lycorine biosynthesis by *Leucojum aestivum* L. and *L. aestivum* ‘Gravety Giant’ plants cultured in bioreactor RITA^®^ . Plant Cell Tissue Organ Cult. 2017, 128, 335–345.

[15] Ivanov, I. , Georgiev, V. , Pavlov, A. Elicitation of galanthamine biosynthesis by *Leucojum aestivum* liquid shoot cultures. J. Plant Physio. 2013, 170, 1122–1129. 10.1016/j.jplph.2013.03.017 23648110

[16] Ptak, A. , El Tahchy, A. , Wyżgolik, G. , Henry, M. et al., Effects of ethylene on somatic embryogenesis and galanthamine content in *Leucojum aestivum* L. cultures. Plant Cell Tiss Organ Cult. 2010, 102, 61–67.

[17] Schumann, A. , Torras‐Claveria, L. , Berkov, S. , Claus, D. et al., Elicitation of galanthamine production by *Leucojum aestivum* shoots grown in temporary immersion system. Biotechnol Prog. 2013, 24, 311–318. 10.1002/btpr.1677 23225790

[18] Ivanov, I. , Georgiev, V. , Georgiev, M. , Ilieva, M. et al., Galanthamine and related alkaloids production by *Leucojum aestivum* L. shoot culture using a temporary immersion technology. Appl. Biochem. Biotechnol. 2011, 163, 268–277. 20680514 10.1007/s12010-010-9036-7

[19] Berkov, S. , Ivanov, I. , Georgiev, V. , Codina, C. , Pavlov, A. Galanthamine biosynthesis in plant *in vitro* systems. Eng. Life Sci. 2014, 14, 643–650.

[20] Georgiev, V. , Ivanov, I. , Berkov, S. , Ilieva, M. et al., Galanthamine production by *Leucojum aestivum* L. shoot culture in a modified bubble column bioreactor with internal sections. Eng. Life Sci. 2012, 12, 534–543.

[21] Ivanov, I. , Georgiev, M. , Georgiev, V. , Ilieva, M. et al., Two‐phase systems for galanthamine biosynthesis: adsorption capacity of resin Amberlite XAD. Scientific work – University Food Technol. 2008, LV, 319–324. (in Bulgarian).

[22] Georgiev, V. , Ivanov, I. , Berkov, S. , Pavlov, A. Alkaloids biosynthesis by *Pancratium maritimum* L. shoots in liquid culture. Acta Physiol. Plant. 2011, 33, 927–933.

[23] Berkov, S. , Bastida, J. , Viladomat, F. , Codina, C. Analysis of galanthamine‐type alkaloids by capillary gas chromatography–mass spectrometry in plants. Phytochem. Anal. 2008, 19, 285–293. 18438759 10.1002/pca.1028

[24] Torras‐Claveria, L. , Berkov, S. , Jauregui, O. , Caujape, J. et al., Metabolic profiling of bioactive *Pancratium canariense* extracts by GC‐MS. Phytochem. Anal. 2010, 21, 80–88. 19774542 10.1002/pca.1158

[25] Marchev A. , Georgiev V. , Ivanov I. , Badjakov I. et al., Two‐phase temporary immersion system for *Agrobacterium rhizogenes* genetic transformation of sage (*Salvia tomentosa* Mill.). Biotechnol. Lett. 2011, 33, 1873–1878. 21516312 10.1007/s10529-011-0625-5

